# Acute Toxicity and Gastroprotective Role of *M. pruriens* in Ethanol-Induced Gastric Mucosal Injuries in Rats

**DOI:** 10.1155/2013/974185

**Published:** 2013-05-28

**Authors:** Shahram Golbabapour, Maryam Hajrezaie, Pouya Hassandarvish, Nazia Abdul Majid, A. Hamid A. Hadi, Noraziah Nordin, Mahmood A. Abdulla

**Affiliations:** ^1^Department of Biomedical Science, Faculty of Medicine, University of Malaya, 50603 Kuala Lumpur, Malaysia; ^2^Institute of Biological Sciences, Faculty of Science, University of Malaya, 50603 Kuala Lumpu, Malaysia; ^3^Department of Chemistry, Faculty of Science, University of Malaya, 50603 Kuala Lumpur, Malaysia; ^4^Department of Pharmacy, Faculty of Medicine, University of Malaya, 50603 Kuala Lumpur, Malaysia

## Abstract

The investigation was to evaluate gastroprotective effects of ethanolic extract of *M. pruriens* leaves on ethanol-induced gastric mucosal injuries in rats. Forty-eight rats were divided into 8 groups: negative control, extract control, ulcer control, reference control, and four experimental groups. As a pretreatment, the negative control and the ulcer control groups were orally administered carboxymethylcellulose (CMC). The reference control was administered omeprazole orally (20 mg/kg). The ethanolic extract of *M. pruriens* leaves was given orally to the extract control group (500 mg/kg) and the experimental groups (62.5, 125, 250, and 500 mg/kg). After 1 h, CMC was given orally to the negative and the extract control groups. The other groups received absolute ethanol. The rats were sacrificed after 1 h. The ulcer control group exhibited significant mucosal injuries with decreased gastric wall mucus and severe damage to the gastric mucosa. The extract caused upregulation of Hsp70 protein, downregulation of Bax protein, and intense periodic acid schiff uptake of glandular portion of stomach. Gastric mucosal homogenate showed significant antioxidant properties with increase in synthesis of PGE2, while MDA was significantly decreased. The ethanolic extract of *M. pruriens* leaves was nontoxic (<5 g/kg) and could enhance defensive mechanisms against hemorrhagic mucosal lesions.

## 1. Introduction

The peptic ulcer, characterized by mucosal damage, is predominantly caused by *Helicobacter pylori*, antiplatelet agents such as acetylsalicylic acid [1], nonsteroidal anti-inflammatory drugs (NSAIDs) such as oral bisphosphonates, potassium chloride, immunosuppressive medications [2, 3], serotonin reuptake inhibitors [4], alcohol consumption, and cigarette smoking (for review, see [5]). Anatomically, the ulcers mostly occur in the stomach and proximal duodenum. In addition, cholinergic hypersensitivity and parasympathetic dominance, as well as gastric hypersecretion, play important roles in peptic ulcer disease. Zollinger-Ellison syndrome, antral G-cell hyperplasia, and bias in the antagonistic gastric hormones are correlated with gastric hypersecretion [2]. The annual incidence rates of uncomplicated and complicated peptic ulcer disease in the general population are approximately one case and 0.7 cases per 1000 people, respectively [6], the majority of whom are below 65 years of age [7]. Symptoms of peptic ulcer disease include abdominal pain, vomiting, and reflux symptoms. Other general symptoms of peptic ulcer disease include loss of appetite and weight [8]. However, younger patients may not show any symptoms. The disease may lead to upper gastrointestinal haemorrhage and perforation [9], which have high morbidity and mortality rates [10]. In the majority of cases, *H. pylori *increases the production of reactive oxygen species (ROS) and reactive nitrogen species (RNS) in the human stomach [11], which induces a regulatory T cell response; the ulcer occurs when the T cell response is inadequate [12]. Furthermore, in association with *H. pylori* that releases ROS, activated neutrophils produce ROS and RNS in the stomach which results in oxidative stress on the gastric mucosa [13]. NSAIDs can cause submucosal erosion and inhibit cyclooxygenase, which reduces the formation of prostaglandins and weakens the protection by the gastric mucosal layer [14, 15]. The basic pathophysiology of gastric ulcers results from imbalance between some endogenous aggressive factor(s) (hydrochloric acid, pepsin, refluxed bile, leukotrienes and ROS) and protective factors, including the function of the mucus-bicarbonate barrier, surface active phospholipids, prostaglandins (PG), mucosal blood flow, cell renewal and migration, nonenzymatic and enzymatic antioxidants, and some growth factors [16, 17]. In spite of the multifaceted pathogenesis of peptic ulcers, secretion of gastric acid is still recognized as a central component of this disease. Therefore, the main therapeutic target is to control acid secretion using antacids, H_2_ receptor blockers (ranitidine and famotidine), or proton pump inhibitors (omeprazole and lansoprazole) [18]. Current gastric ulcer therapies show limited efficacy against gastric mucosal lesions/ulceration and are often associated with several side effects [17]. 

A large number of medicinal plants with gastroprotective properties have been reported by gastric ulcer researchers [19–22]. Plant-based medicines represent a vast untapped source for medicines that have shown enormous therapeutic potential. *M. pruriens*, a tropical legume, is a member of the Fabaceae family and is commonly known as cow itch, Konch, velvet bean, cowage, Bengal bean, Mauritius bean, buffalo bean, kapikacho, and atmagupta. This plant grows 3–18 m in height and is indigenous to tropical regions. Traditionally, it is used in the treatment of some diseases and snake bites as a toxin antagonist, and it is known as an effective aphrodisiac in traditional ayurvedic medicine [23]. It has been shown that the extract of *M. pruriens* is effective on free radical-mediated diseases such as diabetes [24], atherosclerosis [25], and nervous disorders [26], has procoagulant activity [27], and can be used in the management of Parkinson's disease [28]. In addition, *M. pruriens *extract can alleviate male infertility [29, 30] by suppressing psychological stress [29] and improve semen quality through the regulation of steroidogenesis [31]. This extract also has hypocholesterolemic [32], anti-inflammatory, diuretic [33], and antimicrobial activities [34]. Finally, *M. pruriens *extract has been shown to have antioxidant activity [26] to inhibit lipid peroxidation [35] and has positive effects on heart function and the immunological neutralization mechanism. With any etiology, the secretion of gastric acid can increase the incidence of peptic ulcer disease. Maintaining secretion at a normal level is the main therapeutic target. The combination of antiacids, antisecretory drugs, and antimicrobial agents has been suggested for peptic ulcer treatment. A combination treatment of H_2_ receptor antagonists such as the H_2_RAs cimetidine and ranitidine [36], proton pump inhibitors [37], and clarithromycin, amoxicillin, or metronidazole [34] serves as the routine therapeutic action. Although various endoscopic and pharmacological therapies are available for peptic ulcer disease, these treatments mostly show limited efficacy against gastric diseases and are often associated with severe side effects [38]. In contrast, natural products have shown effective therapeutic properties with reduced side effects [39–41], and several studies have been performed in this field [42, 43]. In addition, the pharmaceutical industry has become more inclined to investigate the advantages of herbal therapeutics [44]. Among a variety of tropical plants, *M. pruriens* shows fascinating therapeutic properties, especially the antimicrobial effects that make this plant a candidate for further research on its effects on peptic ulcer disease. The present study was undertaken to investigate the mechanisms of the gastroprotective properties of the ethanolic extract of *M. pruriens* leaves on ethanol-induced gastric mucosal injury in rats.

## 2. Materials and Methods

### 2.1. Animal Experimentation

Animal care and experimental procedures were performed in accordance with the Guide for the Care and Use of Laboratory Animals (National Institute of Health) with approval from the committee for animal experimentation—Faculty of Medicine, University of Malaya (University of Malaya—Ethic no. (ISB/30/05/2012/SG (R))).

### 2.2. Omeprazole

Omeprazole was used as a reference gastroprotective drug and was obtained from the University of Malaya Medical Centre (UMMC) Pharmacy. The drug was suspended in 0.5% (w/v) carboxymethylcellulose (CMC) and administered orally to the rats at a dosage of 20 mg/kg body weight (5 mL/kg), recommended by various researchers [45, 46].

### 2.3. Plant Specimen and Preparation of Extraction

Fresh *M. pruriens *leaves were obtained from Ethno Resources (Selangor, Malaysia) with the approved voucher specimen (KLU 45415-Herbarium of RimbaIlmu, Institute of Biological Sciences, University of Malaya). The dried leaves were ground into fine powder by an electrical blender. The powder was soaked in 95% ethanol (100 g/500 mL) for 3 days and was filtered through both a fine muslin cloth and a filter paper (Whatman No. 1). Then, it was distilled (11.3% dried mass) with a rotary evaporator (Eyelet, USA). The dried extract was dissolved in CMC for the oral administration at a dosage of 62.5, 125, 250, or 500 mg/kg body weight (in 0.5% CMC, 5 mL/kg body weight).

### 2.4. Acute Toxicity Test and Experimental Animals

Healthy male and female *Sprague Dawley *rats (6–8 weeks old, weighed between 150 and 200 g) were obtained from the Animal House, University of Malaya. The animals were given standard rat pellets and tap water *ad libitum*, individually caged in a wide-mesh wire bottom type to prevent coprophagia. The acute toxicity study was used to determine a safe dose for the *M. pruriens* extract. The rats (18 males and 18 females) were assigned equally into 3 groups; vehicle (0.5% CMC, 5 mL/kg), 2 g/kg and 5 g/kg of the leaf extract (5 mL/kg). Prior to the dosing, the animals were fasted overnight (food but not water). Food was withheld for a further 3 to 4 h after dosing. The animals were observed for 48 h after the administration of the powder for the onset of clinical or toxicological symptoms. Mortality, if any, was reported over a period of 2 weeks. The animals were sacrificed then by giving an overdose of xylazine and ketamine anaesthesia on the 15th day. Histological and serum biochemical parameters were determined following standard methods. 

### 2.5. Experimental Animals for Gastric Ulcer Studies

Healthy adult *Sprague Dawley *rats, weighed between 200 and 250 g, were obtained from the Experimental Animal House, University of Malaya. The rats were randomly divided into 8 groups of 6 rats each and placed individually in a cage with wide-mesh wire bottom to prevent coprophagia. The animals were maintained on a standard pellet diet and tap water *ad libitum*. 

### 2.6. Gastric Ulcer Induction by Ethanol

Animals were fasted for 24 h prior to the experiment. Drinking was allowed up to 2 h before the experiment. The negative control group (group 1) and the ulcer control group (group 3) received the vehicle (0.5% CMC) orally. The extract control group (group 2) received 500 mg/kg of the *M. pruriens* extract orally. The reference group (group 4) received an oral dose of 20 mg/kg omeprazole in 0.5% CMC (5 mL/kg), and the experimental groups received the ethanolic extract of *M. pruriens* at a single dose of 62.5, 125, 250, or 500 mg/kg (groups 5–8, resp.). All of these dosages were administered as pretreatments. 1 h after the pretreatments, the vehicle was administrated to the group 1 and 2. Absolute ethanol was orally administered to the other groups. After 60 min, the rats were euthanized (over dose of xylazine and ketamine), and their stomachs were dissected.

### 2.7. Macroscopic Gastric Lesion Evaluation

The gastric mucosa was also examined for damage with a stereomicroscope. Dimensions of each individual hemorrhagic lesion were measured by a planimeter (10 × 10 mm^2^ = ulcer area (UA)) under a stereomicroscope (1.8x). The ulcers mostly appeared parallel to the long axis of the stomach. The number of small squares, 2 mm × 2 mm, covering the length and width of each ulcer band was determined. The UA was calculated according to the previously published protocol [47]; the UA, in square millimeters, was calculated through the following formula:

(1) $$\begin{array}{c} \text{Sum}\;\text{of}\;\text{small}\;\text{squares}\times 4\times 1.8=\text{UA}\;\left({\text{mm}}^{2}\right). \end{array}$$

Inhibition percentage (I%) was calculated as follows:

(2) $$\begin{array}{c} \left(\text{I}\%\right)=\left[\left({\text{UA}}_{\text{control}}-{\text{UA}}_{\text{treated}}\right)÷{\text{UA}}_{\text{control}}\right]\times 100\%. \end{array}$$

### 2.8. Determination of Gastric Wall Mucus

The gastric wall mucus was evaluated according to the modified procedure of Corne et al. [48]. Glandular stomach segments were separated from the lumen. Then each segment was transferred immediately to 10 mL of 0.1% w/v alcian-blue solution in a 0.16 M sucrose solution buffered with 0.05 M of sodium acetate at pH 5 [49]. The tissue was stained for 2 h in alcian blue. Excess dye was removed with 2 successive rinses of 10 mL of 0.25 M sucrose. Dye was mixed with gastric wall mucus and was extracted with 10 mL of 0.5 M magnesium chloride by intermittently shakings for 1 min at a 30 min interval for 2 h. Then the blue extract was shaken with diethyl ether. The resulting emulsion was centrifuged at 3000 rpm for 10 min, and the absorbance of the aqueous layer was recorded at 580 nm. The quantity of alcian blue extracted in wet glandular tissue (mg alcian-blue/g tissue) was then measured.

### 2.9. Assays for Bioactivities

Gastric tissue samples were washed thoroughly with ice-cold saline. A homogenate (10% (w/v)) was prepared on ice cold with phosphate-buffered saline (PBS); buffer (a 50 mM phosphate buffer, pH 7.4) contained a mammalian protease inhibitor cocktail. The homogenate was centrifuged at 4,000 rpm for 10 min (4°C). The supernatant was used to measure activities of nitric oxide (NO), catalase (CAT), superoxide dismutase (SOD), glutathione (GSH), prostaglandin E2 (PGE2), and malondialdehyde (MDA). These assays were performed according to the respective manufacturer protocols (Cayman, USA). Total protein content of the supernatant was determined by the Bio-Rad protein assay kit (Bio-Rad, USA) using bovine serum albumin as the standard.

Nitric oxide (NO) content was measured through nitrite/nitrate concentration [50], using Griess reagent (Sigma, USA). In brief, Griess reagent (0.1% N-(1-naphthyl) ethylenediamidedihydrochloride, 1% sulfanilamide in 5% phosphoric acid) was added to the supernatant (1 : 1). The optical density was measured at 540 nm after 10 min. Sodium nitrite was used as a standard. 

Using the SOD assay kit, xanthine oxidase and hypoxanthine detected superoxide radicals. In brief, the kit could measure the amount of enzyme that caused 50% dismutation of the superoxide radical. The SOD activity (Cu/Zn, Mn and FeSOD) of the supernatants (10 *μ*L) was diluted with the tetrazolium salt solution (200 *μ*L). The reaction was initiated by adding xanthine oxidase (20 *μ*L) and incubated for 20 min. The absorbance was read at 450 nm. In this assay bovine erythrocyte SOD (Cu/Zn) was used as the standard. 

In this study GSH was quantified using glutathione reductase. In brief, sample supernatant (50 *μ*L) was added to the assay cocktail that contained 0.4 M 2-(N-morpholino) ethanesulfonic acid, 0.1 M phosphate, 2 mM EDTA, reconstituted NADP+ and glucose-6-phosphate and reconstituted glutathione reductase, and glucose-6-phosphate dehydrogenase. In this assay GSSG, produced during the reduction of hydroperoxides by glutathione peroxidase, was used to create the standard curve. 

Production of PGE2 in the tissue homogenate supernatant was determined using enzyme immunoassay according to the manufacturer's instructions. Briefly, the kit converted PGE2 into Bicyclo PGE2 (stable derivative) which was measurable by the kit. The tissue supernatant and standards were added to a 96-well plate, precoated with goat polyclonal anti-mouse IgG. After incubation period with PGE2 acetylcholinesterase conjugated with the PGE2 Tracer, Ellman's reagent was applied for 60 min which The product of this enzymatic reaction had a distinct yellow colour and absorbs strongly at 412 nm. Results were calculated using the standard curve which was expressed as picogram per milliliter (pg/mL).

The concentrations of gastric mucosal lipid peroxidation were determined by estimating MDA using the thiobarbituric acid test [51]. In brief, SDS solution (100 *μ*L), the supernatant (100 *μ*L), and color reagent (4 mL) were mixed in a vial and incubated for 1 h (100°C), followed by an incubation on ice for 10 min. Then the vials were centrifuged at 1,600 ×g (for 10 min, at 4°C). Within 30 min, 150 *μ*L of each vial was placed on a 96-well plate and the absorbance was read at 530–540 nm.

### 2.10. Histological Studies of the Gastric Mucosa

#### 2.10.1. Preparation of Tissue Sections

Specimens of the gastric walls were fixed in 10% buffered formalin for 18 h at 4°C and were processed using the paraffin tissue-processing machine (Leica, Germany). Sections of the stomach were made at a thickness of 5 *μ*m (Leica Rotation Microtome, Germany).

#### 2.10.2. Hematoxylin and Eosin

Stomach section was stained with hematoxylin and eosin for histological evaluation [52]. 

#### 2.10.3. Study of Mucosal Glycoproteins

Sections of 5 *μ*m thickness of the glandular portion of stomach were stained with periodic acid schiff (PAS) to visualize mucus production and changes in both acidic and basic glycoproteins [53].

#### 2.10.4. Immunohistochemical Staining

Tissue section of 5 *μ*m in thickness was cut from each block and then deparaffinized and dehydrated. The tissue sections was then placed on 3-aminopropyltrimethoxysilane (APES)-treated glass slides. The tissue section slides were heated with a hot air oven (Venticell, Germany), at 60°C for 20 min. The tissue sections were deparaffinised, rehydrated, and stained according to the manufacturer's protocol. Antigen retrieval process was performed in 10 mM sodium citrate buffer. Immunochemical staining Hsp70 (Abcam, USA) and Bax (Abcam, USA) were performed using a streptavidin peroxidase (Abcam, USA) procedure. Briefly, tissue sections were washed with the washing buffer and incubated (15 min) with the biotinylated primary antibody; HSP70 (1 : 500) and Bax (1 : 200). The sections were with the washing buffer. In a humidified chamber, sufficient amount of streptavidin HRP (streptavidin conjugated with horseradish peroxidase in PBS) was added and incubated for 15 min. Then the tissue sections were washed with the washing buffer. For 5 min diaminobenzidine (DAB) substrate chromogen was applied to the tissue sections. The sections were washed with the buffer and counterstained with hematoxylin for 5 seconds. The sections were then dipped in weak ammonia (0.037 M/L) 10 times and then rinsed with distilled water and coverslipped. Positive findings of the immunohistochemical staining should appear as brown stains under light microscope.

### 2.11. Statistical Analysis

All values were reported as mean ± S.E.M. The statistical significance of differences between groups was assessed using one-way ANOVA (post hoc analysis). A value of *P* < 0.05 was considered significant.

## 3. Results

### 3.1. Acute Toxicity Study

Acute toxicity study did not show any sign of toxicity. There was no histological sign of hepatic toxicity and renal toxicity. Moreover, blood biochemistry analysis appeared normal (see Figure S1 and Table S1 in Supplementary Material available online at http://dx.doi.org/10.1155/2013/974185).

### 3.2. Gross Evaluation of Gastric Lesions

**Figure 1:**
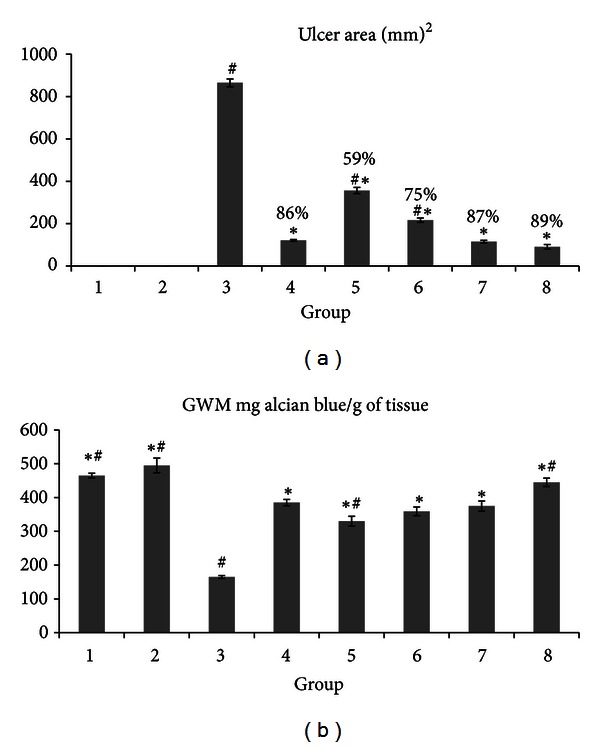
Effects of *M. pruriens* extract on ulcer area (mm^2^) and inhibition percentage (a) and alcian-blue-binding capacity (b). Ulcer area (mm^2^) and inhibition percentage and inhibition of the gastric lesions (%) are indicated in brackets above the columns. Alcian-blue-binding capacity is defined as gastric wall mucus (GWM). Gastric wall mucus groups 1, 2, 3, and 4 represent the negative control, the extract control, the ulcer control, and the reference groups, respectively. The experimental groups are presented as groups (groups 5–8). All values are expressed as mean ± standard error mean. All values are expressed as mean ± standard error mean. Mean difference is significant at the *P* < 0.05 level (one way between groups ANOVA with post hoc analysis). *Significant when compared with the group 3. ^#^Significant when compared with the group 4.

**Figure 2:**
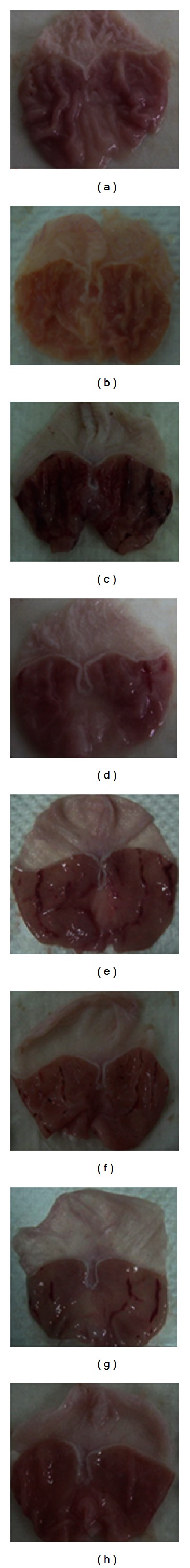
Effects of *M. pruriens* extract on macroscopic appearance of the gastric mucosa. The negative control group and the extract control group show no injuries to the gastric mucosa ((a) and (b)). Severe injuries are observed in the gastric mucosa of the ulcer control group. Ethanol produced extensive visible hemorrhagic necrosis of the gastric mucosa (c). The reference control group, pretreated with omeprazole (20 mg/kg), shows mild injuries to the gastric mucosa (d). In the experimental groups, rats pretreated with 62.5 mg/kg of the extract shows moderate injuries in the gastric mucosa (e); in the pretreatment with 125 mg/kg of the extract, more moderate injuries are observed in the gastric mucosa (f); rats pretreated with 250 show mild injuries in the mucosa (g); pretreatment with 500 mg/kg of the extract shows no injuries to the gastric mucosa; instead, flattening of the gastric mucosa is observed (h).

The gastroprotective activity of the *M. pruriens* leaf extract in the ethanol-induced gastric lesion model is shown in Figure 1(a). The results showed that rats pretreated with omeprazole (group 4) or *M. pruriens *extracts (groups 5–8) before being given absolute ethanol had significantly reduced areas of gastric ulcers as shown in the ulcer group (Figures 1(a) and 2). Absolute ethanol produced extensive and visible hemorrhagic lesions in the gastric mucosa. In a dose-dependent manner, *M. pruriens* significantly inhibited ulcer formation induced by absolute ethanol and obviously decreased the gastric mucosal damage (Figure 2) indicating that the *M. pruriens* extract significantly suppressed the formation of ulcers. Intriguingly, among the experimental groups, the dose of 500 mg/kg showed the flattening of the gastric mucosal folds (Figure 2(h)). It was also evidenced that the protection of the gastric mucosa was most prominent in this dosage (Figure 1(a)). The significant inhibition of gastric ulcer formation in the dosage of 250 mg/kg (group 7) was comparable to the inhibition observed in group 4 (Figures 1 and 2). The extract by itself did not show any sign of abnormality in group 2 (Figures 1(a) and 2(b)). 

### 3.3. Effect of *M. pruriens* on Gastric Wall Mucus

Figure 1(b) illustrates that group 3 significantly decreased alcian-blue-binding capacity of the gastric wall mucus, compared to the negative control group. Unsurprisingly, the experimental groups showed significant enhancement of the alcian-blue-binding capacity of the gastric mucosa. The level of alcian-blue-binding capacity in group 3 showed a meaningful difference in comparison to the negative control group. The binding capacity appeared bias to the extract control (Figure 1(b)).

### 3.4. Effect of *M. pruriens* on Antioxidant Activity

Table 1 shows that antioxidant activities of the tissue homogenates were remarkably different among groups. The activity level of NO in those groups pretreated with CMC (group 1 and group 2) was the highest while that of group 3 was the lowest. Among those rats receiving ethanol, group 4 and group 6 confirmed the effect of omeprazol and the plant extract on enhancement of activity of NO. In parallel, the activity of CAT in tissue homogenates showed the highest activity in group 8 (Table 1). In group 2, ethanol reduced activities of SOD and GSH compared to the negative control group. The experimental groups had significant increase in the enzyme activities compared with group 3 (Table 1). These assays showed almost the same level of the activities between group 1 and group 2.

### 3.5. Effect of *M. pruriens* Extract on PGE2

In this study effect of the *M. pruriens *extract on synthesis of PGE2 in gastric mucosal homogenates was assessed. Ethanol in the ulcer control group significantly caused depletion of PGE2 compared to groups 1 and 2. The experimental groups on the other hand showed significantly augmented PGE2 content (Table 2).

### 3.6. Effect of *M. pruriens* Extract on Tissue MDA

MDA activity was significantly increased in group 3 compared to group 1. The extract control group showed the lowest activity of MDA. Expectedly, the experimental groups showed significantly decrease in MDA activity (Table 2).

### 3.7. Effect of *M. pruriens* Extract on Protein Concentration

Protein concentration of the gastric mucosal homogenate in the ulcer control group was significantly decreased compared with that in group 1, which was nearly the same as the extract control group. Groups 5–8, compared to the ulcer control group, exhibited significantly increased protein concentrations (Table 2).

### 3.8. Histological Evaluation of Gastric Lesions

**Figure 3:**
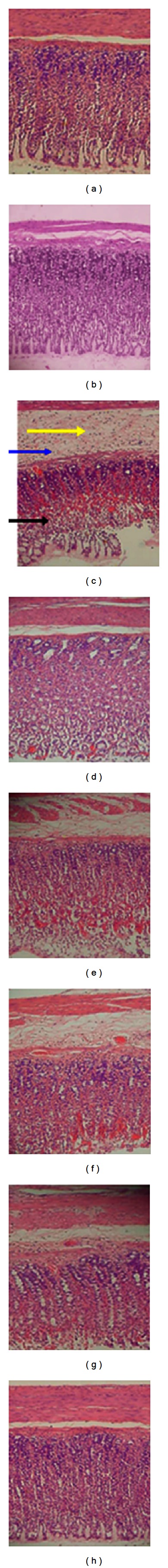
Histological effects of *M. pruriens* on gastric tissue (H&E staining 20x). In the negative control group and the extract control group no injuries to the gastric mucosa are observed ((a) and (b)). The ulcer control group has severe disruption to the surface epithelium (black arrow); necrotic lesions penetrating deeply into the mucosa, extensive edema of the submucosal layer (yellow arrow) and leucocyte infiltration (blue arrow) (c). The reference control group shows mild disruption of the surface epithelium mucosa. There are edema and leucocyte infiltration of the submucosal layer (d). In the experimental groups, rats pretreated with 62.5 mg/kg of the extract show moderate disruption of the surface epithelium with edema and leucocytes infiltration of the submucosal layer (e); in the pretreatment with 125 mg/kg of the extract, rats showed a mild disruption of the surface epithelium with edema and leucocyte infiltration in submucosal layer (f); rats pretreated with 250 shows mild disruption of the surface epithelium with edema and leucocytes infiltration of the submucosal layer (g); pretreatment with 500 mg/kg of the extract showed mild edema and leucocytes infiltration of the submucosal layer but no disruption of the surface epithelium (h).

Histological observation showed extensive damage of the gastric mucosa in the ulcer control group (Figure 3). Necrotic lesions penetrated deeply into the mucosa, and extensive edema and leukocyte infiltration of the submucosal layer are presented (Figure 3). Rats in the experimental groups had comparatively better protection of the gastric mucosa, evidenced by the reduction or absence of the ulcer area, submucosal edema, and leukocyte infiltration (Figure 3). The plant extracts exerted protective effects in a dose-dependent manner. Histological evaluation of the extract control group did not show any meaningful changes compared to group 1 (Figure 3).

### 3.9. Periodic Acid Schiff of Mucosal Glycoproteins

**Figure 4:**
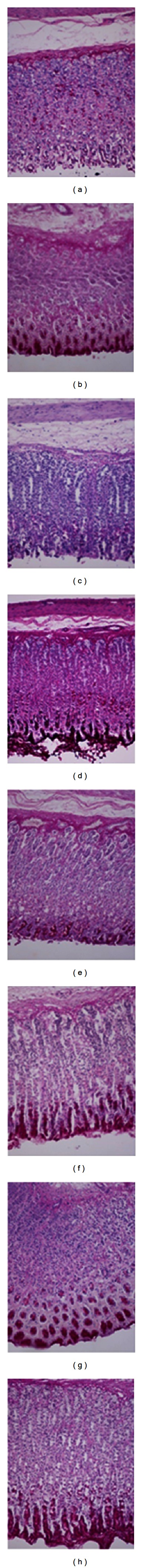
Effects of *M. pruriens* on gastric tissue glycoprotein (PAS staining 20x). The negative control group (a); the extract control group (b); the ulcer control group (c); the reference control group (d); the experimental groups pretreated with 62.5, 125, 250, and 500 mg/kg ((e), (f), (g), and (h) resp.). Magenta color in the apical epithelial cells in the pretreated groups with the extract ((e), (f), (g), and (h)) shows gradual increase in mucosal secretion of gastric glands. The intense secretion of mucus in gastric glands is demonstrated in the extract control group and the experimental group pretreated with 500 mg/kg of the extract ((b) and (h)).

The gastric mucosa in the rats pretreated with omeprazole or *M. pruriens *extract (groups 5–8) showed increase in PAS staining intensity compared to groups 1–3 (Figure 4), indicating increased glycoprotein content of gastric mucosa in pretreated rats. The intensity of PAS staining was comparatively more in the negative control group than in the ulcer control group. Groups 5–8 along with the reference group reversed the decrease in PAS staining caused by absolute ethanol (Figure 4). Group 2 exhibited high intensity of PAS staining when compared to group 1. The intensity was comparable to that of the experimental groups that received 250 mg/kg (group 7) of the extract. 

### 3.10. Immunohistochemistry

**Figure 5:**
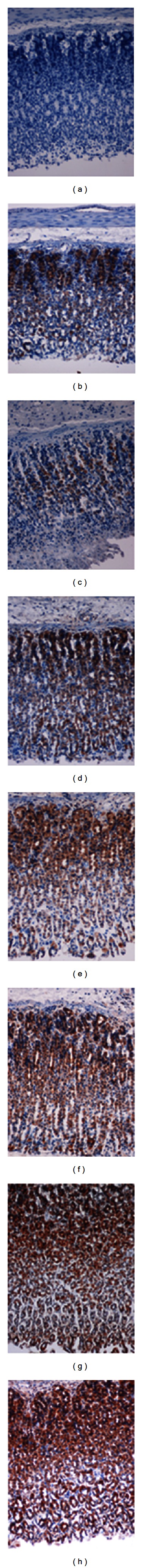
Immunohistochemical expression of Hsp70 in the gastric mucosa (20x). The negative control group (a); the extract control group (b); the ulcer control group (c); the reference control group (d); the experimental groups pretreated with 62.5, 125, 250, and 500 mg/kg ((e), (f), (g), and (h) resp.). Upregulation of Hsp70 protein in rats pretreated with omeprazole (d) or *M. pruriens *extract ((b) and (e)–(h)) compared with the ulcer control (c).

**Figure 6:**
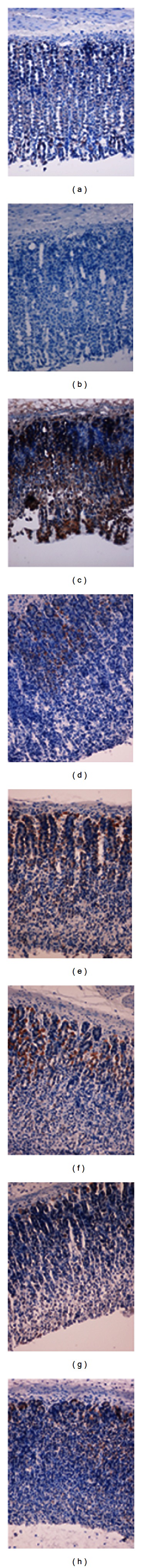
Immunohistochemical expression of Bax in the gastric mucosa (20x). The negative control group (a); the extract control group (b); the ulcer control group (c); the reference control group (d); the experimental groups pretreated with 62.5, 125, 250, and 500 mg/kg ((e), (f), (g), and (h) resp.). Immunohistochemical analysis of Bax protein showed downexpression of Bax protein in rats pretreated with *M. pruriens *extract ((b) and (e)–(h)).

Immunohistochemical staining of Hsp70 protein of gastric mucosa in rats pretreated with *M. pruriens *extract or omeprazole (the experimental groups or the reference group, resp.) showed upregulation of Hsp70 protein compared to the ulcer control group (Figure 5). The extract control group exhibited upregulation of Hsp70 protein compared to group 1. Comparison between immunohistochemical staining of Bax protein of gastric mucosa of the same groups showed downregulation of Bax protein (Figure 6). In addition, the expression of Bax protein in the ulcer control group was found upregulated compared to group 4–8 (Figure 6). The expression of Bax protein in group 1 and group 2 was found similar.

## 4. Discussion

The acute toxicity test did not show any signs of toxicity or mortality. This test revealed that the plant is safe and has no toxicity when administered orally up to 5 g/kg. Ethanol intoxication caused significant decreases in the protein concentrations in the ulcer control group compared to the other groups. It was shown that absolute ethanol damages epithelial cells, which leads to a reduction in protein concentration [54]. The mucus membrane acts as the first layer of defense for stomach tissue and was also eroded by ethanol. The gastric mucosa prevents contact between the stomach wall and digestive enzymes, such as pepsin [54]. Similarly, this study showed that the administration of ethanol reduced the protein content. Pretreatment with the extract enhanced the generation of epithelial cells, which gave rise to a significant increase in the protein concentration in the gastric secretions of the pretreated group. This observation was confirmed when the protein contents of both the normal control group and the extract control group appeared in almost the same range. 

Imbalance between the protective and the aggressive mechanisms of the mucosa, which may be triggered by several endogenous factors and aggressive exogenous factors, is the main cause of peptic ulcer [55]. Alcohol-induced gastric lesions impair gastric defensive factors such as mucus and mucosal circulation [56]. Ethanol causes necrotic lesions of the gastric mucosa in a multifaceted manner. Ethanol produces necrotic lesions by direct necrotizing action, which in turn reduces defensive factors, including the secretion of bicarbonate and production of mucus [57]. However, omeprazole, besides its antisecretory effect and effectiveness in acid-dependent ulcer models, is also effective in acid-independent models, like ethanol-ulcer model, exert mucosal protection and in non-anti-secretory doses [58, 59]. Similarly, H_2_ blocking drugs can also induce gastroprotection in non-antisecretory doses [60]. Our results showed that absolute ethanol extensively damaged the gastric mucosa, leading to increased neutrophil infiltration into the gastric mucosa. Activation and infiltration of neutrophils appeared to be involved in the initial processes of formation of the lesion. The gastric wall mucus is an important defensive barrier against gastrointestinal damage [61]. Mucus secretion is regarded as a crucial defensive factor in the protection of the gastric mucosa from gastric lesions [62]. The level of gastric wall mucus was previously determined and was used as an indicator for gastric mucus secretion. The finding that pretreatment with the *M. pruriens *extract significantly increased the gastric mucus content in rats with ethanol-induced ulcers suggested that the gastroprotective effect of *M. pruriens *was mediated partly by preservation of the gastric wall mucus. On the other hand, some of the commercial antiulcer medicines increase the amount of gastric mucus secretion of the gastric mucosa [63]. Gastric mucus consists of mucin-type glycoproteins, which can be detected by alcian-blue [64]. Gastric wall mucus depletion induced by ethanol is also one of the pathogenic mechanisms responsible for gastric lesions [65]. Alcian-blue dye binds to negatively charged materials. The increase in bound alcian blue indicates the protective effect of orally administered *M. pruriens*, which may occur through the formation of protective complexes between *M. pruriens *and the mucus that acts as a barrier against several necrotizing agents introduced in the stomach [66]. In the experimental groups, *M. pruriens *replenished the decreased concentration of gastric wall mucus that was reduced by ethanol. Thus, a possible mechanism for gastric mucosal protection of *M. pruriens* is the reinforcement of the resistance of the mucosal barrier through a protective coating which has an important role in preventing gastric tissue from damages and in facilitating the repair of the gastric epithelium [67]. Constantly, the level of alcian-blue-binding capacity in the extract control group showed that the plant extract by itself increased the secretion of gastric wall mucosa. 

The preservation of adherent mucus on the glandular mucosa is one of the contributing factors in the prevention of gastric mucosal damage induced by chemical irritants [67]. Therefore, it seems probable that the protective effect of *M. pruriens* is partly due to the preservation of the mucus layer in the gastric mucosa. This study showed that* M. pruriens* prevented ethanol-induced gastric wall mucus depletion. 

The effects of absolute ethanol on gastric mucosa stimulate biological reactions in the cells, such as lipid peroxidation, formation of free radicals, intracellular oxidative stress, changes in permeability and depolarization of the mitochondrial membrane, and eventually cell death. Oral administration of absolute ethanol to the rats caused linear hemorrhagic lesions, extensive submucosal edema, mucosal friability, inflammatory cell infiltration, and epithelial cell loss in the stomach, which were the typical characteristics of alcohol injury [68]. The pathogenesis of ethanol-induced gastric mucosal damage occurs directly and indirectly through various biomediators, such as lipoxygenase, cytokines, and oxygen-derived free radicals. The effect of *M. pruriens* on gastric wall mucus showed that the plant extract increased the mucus of the gastric wall, consistent with results reported by Thirunavukkarasu et al. [69]. 

Omeprazole, a proton pump inhibitor (PPI), exhibits an antisecretory and protective effect [70]. This medicine is widely used as an acid inhibitor agent for the treatment of disorders related to gastric acid secretion [71]. Omeprazole is effective in treating peptic ulcer disease and gastroesophageal reflux in short-term and long-term use [72]. PPIs are able to cause a relatively complete suppression of acid secretion. Absolute ethanol extensively damages the gastric mucosa and leads to increased neutrophil infiltration into the gastric mucosa. Neutrophils mediate lipid peroxidation through the production of superoxide anions. This study revealed that *M. pruriens* extract had a protection effect on gastric mucosa, inhibited leukocyte infiltration to the gastric wall in the pretreated rats, and caused a reduction of neutrophil infiltration into ulcerated tissue. The activation and infiltration of neutrophils appeared as a key factor in initial processes of gastric lesions formation. Several studies showed that reduction of neutrophil infiltration into ulcerated gastric tissues promoted the prevention of gastric ulcers in rats [40–43]. 

Another gastroprotective mechanism of the *M. pruriens* leaf extract might be due to a decrease in gastric motility leading to more flattened gastric surface. Changes in gastric motility play an important role in the development and prevention of experimental gastric lesions [73]. The relaxation of the circular muscles may protect the gastric mucosa through the flattening of the folds [19, 74, 75]. This flattening will increase the mucosal area exposed to necrotizing agents and reduce the volume of the gastric irritants on the rugal crest [19, 76].

The gastric tissue homogenate of the experimental groups showed significant antioxidant activity through the increase of antioxidant reactions (NO, CAT, SOD, and GSH) and PGE2 and the decrease of MDA level in response to oxidative stress induced by absolute ethanol. The extract by itself did not change antioxidant activities significantly, however, it caused a significant reduction in the MDA level. SOD converts superoxide to H_2_O_2_, which in turn converts to water by CAT in lysosomes or by glutathione peroxidase in mitochondria [77]. MDA is the final product of lipid peroxidation and is an indicator to measure lipid peroxidation [78]. Gastric MDA increased in the ulcer control group and decreased in the experimental groups. Lipid peroxidation causes loss of membrane fluidity, impaired ion transport and membrane integrity and finally loss of cellular functions. Stress also causes the inactivation of prostaglandin synthetase leading to decreased biosynthesis of prostaglandin, the key factor in gastroprotection against most mucosa irritation. Moreover, PGE2 plays an important role in the regulation of gastric mucus secretion [79]. PGE2 has protective effects on various gastric injury models [80]. Ethanol reduces the mucosal PGE2 content [81]. PGE2 exerts a protective action on the stomach through the activation of prostaglandin E receptors [82]. Consistent with previous studies [42, 43], this study showed that the mucosal level of PGE2 showed that biosynthesis of PGE2 enhanced in the experimental groups, suggesting that the gastroprotective effect of the plant extract was mediated partially by PGE2. The plant extract alone showed the same level of PGE2. Prostaglandins potentially influence every component of the mucosal defense: stimulating mucus and bicarbonate secretion, maintaining mucosal blood flow, enhancing the resistance of epithelial cells to injury induced by cytotoxins, and inhibiting leukocyte recruitment [83]. Prostaglandins exert a gastroprotective action against gastric mucosal injuries through maintenance of gastric mucus synthesis and secretion [84].

PAS histochemical method exhibits characteristic carmine staining of stomach regions that secrete mucopolysaccharides. Among the experimental groups, rats pretreated with 500 mg/kg of the extract showed intense secretion of mucus in gastric glands. Mucus production represents one of the main mechanisms of local gastric mucosal defense [85]. A number of factors appear to influence gastric ulcer prevention, but mucus and bicarbonate secretion may be the most effective process in the ulcer prevention as the mucus/bicarbonate layer protects newly formed cells from acid and peptic injury [86]. The extract by itself increased intense secretion of mucus compared to the normal control group. This finding emphasised on the ability of the extract to imply mucus secretion.

Hsp70 is a 70 kDa protein from the Hsp family present on mammalian cells. It is the most conserved and abundantly produced protein in response to different forms of stress [87], such as heat, toxic agents, infection, and proliferation [88]. Bax (a proapoptotic protein) promotes apoptosis [89]. The susceptibility of a cell to apoptosis depends on the balance between apoptosis-promoting and apoptosis-suppressing factors [90]. These proteins are responsible for protecting cellular homeostatic processes from environmental and physiologic injuries by preserving the structure of normal proteins and repairing or removing damaged proteins [91], which makes the study of this protein an interesting element for possible mechanisms of action elucidation. Hsp70 proteins defend cells from oxidative stress or heat shock. Ethanol-generated ROS normally act to inhibit the expression of Hsp70 and increase the expression of Bax. Hsp70 prevents these partially denatured proteins from aggregating and allows them to refold. As previous studies reported that the overexpression of Hsp70 could protect stomach [40–43], this study showed that the plant extract protected the gastric tissues through upregulation of Hsp70. Our results showed significant expression of Hsp70 in the extract control group and the experimental group. Hsp70 has been suggested to exert its gastroprotective action through protecting mitochondria and through interfering with the stress-induced apoptotic program [92]. Immunohistochemical staining of Bax protein of gastric mucosa in rats pretreated with *M. pruriens* extract or omeprazole showed downregulation of Bax protein. Similar to the previous studies [40–43], the expression of Bax protein in the ulcer control group was found upregulated compared to the groups pretreated either with omeprazole or with plant extract. 

## 5. Conclusion

Acute toxicity study demonstrated that rats treated with the *M. pruriens *(up to 5 g/kg) manifested no abnormal signs. This plant could significantly protect the gastric mucosa against ethanol-induced injury. Such protection was ascertained macroscopically by significant increase in the gastric wall mucus in comparison with the ulcer control group. Also the reduction of ulcer areas in the gastric wall as well as the reduction or inhibition of edema and leukocytes infiltration of the submucosal layers was shown histologically. Rats pretreated with the plant extract showed upregulation of Hsp70 protein and downregulation of Bax protein. Increase in the PAS staining of gastric mucosa of the pretreated rats with the extract indicated an increase in glycoprotein content. *M. pruriens *reversed the decrease in PAS staining induced by ethanol. Bioactivity of gastric tissue homogenates revealed that this plant significantly increased the PGE2 and SOD and decreased the level of MDA in the respective pretreated groups. This study provided evidence that the *M. pruriens *possessed a gastroprotective effect, which appeared partly due to the preservation of gastric mucus secretion, increased production of Hsp70 protein, and the antioxidant enzymes. 

## Supplementary Material

suppl M. pruriens extract (<5 g/kg) in the acute toxicity test, on Sprague Dawley rats (6-8 weeks old, male and female), did not show any sign of toxicity in histological sections (H&E staining) of liver and kidney and in serum biochemical parameters.

## Figures and Tables

**Table 1:** Antioxidant activities of tissue homogenates.

Groups	NO (*µ*M)	CAT nM/min/mL	SOD (U/g protein)	GSH (*µ*M/mg protein)
1	9.16^∗#^ ± 0.04	126.66* ± 1.86	472.58^∗#^ ± 4.98	16.33^∗#^ ± 0.07
2	9.14^∗#^ ± 0.18	133.68* ± 1.90	496.20^∗#^ ± 4.63	15.67^∗#^ ± 0.19
3	3.64^#^ ± 0.14	71.20^#^ ± 2.88	170.43^#^ ± 7.49	10.28^#^ ± 0.43
4	7.65* ± 0.20	129.83* ± 5.13	395.14* ± 6.03	14.48* ± 0.13
5	5.31^∗#^ ± 0.10	91.18^∗#^ ± 3.02	300.51^∗#^ ± 6.49	11.51^∗#^ ± 0.13
6	7.19* ± 0.03	102.35^∗#^ ± 3.38	348.12^∗#^ ± 4.75	11.95^∗#^ ± 0.21
7	7.06^∗#^ ± 0.11	109.61^∗#^ ± 5.02	367.81^∗#^ ± 4.30	13.17^∗#^ ± 0.13
8	6.15^∗#^ ± 0.03	133.71* ± 1.56	387.37* ± 3.34	13.27^∗#^ ± 0.23

NO: nitric oxide; SOD: superoxide dismutase; GSH: glutathione. Groups 1 to 4 represent the negative control group, the extract control group, the ulcer control group, and the reference control group, respectively. The experimental groups are presented as groups 4–8. All values are expressed as mean  ±  standard error mean. Mean difference is significant at the *P* < 0.05 level (one-way between groups ANOVA with post hoc analysis). *Significant when compared with the group 3. ^#^Significant when compared with the group 4.

**Table 2:** Enzymatic activities and protein concentration of tissue homogenates.

Groups	PGE2 (ng/mg protein)	MDA (*µ*M/g protein)	Protein concentration (mg/mL tissue)
1	565.38^∗#^ ± 6.51	104.18* ± 2.03	14.13^∗#^ ± 0.15
2	572.03^∗#^ ± 5.72	105.71* ± 3.02	14.55^∗#^ ± 0.12
3	72.50^#^ ± 0.593	230.05^#^ ± 1.04	7.13^#^ ± 0.14
4	301.70* ± 3.38	109.12* ± 0.97	12.34* ± 0.11
5	234.93^∗#^ ± 4.70	101.01* ± 1.54	10.26^∗#^ ± 0.11
6	257.34^∗#^ ± 2.25	101.82* ± 1.51	11.02^∗#^ ± 0.12
7	271.11^∗#^ ± 4.58	104.58* ± 2.17	11.44^∗#^ ± 0.12
8	296.67* ± 3.91	108.81* ± 1.41	11.70^∗#^ ± 0.10

PGE2: prostaglandinE2; MDA: malondialdehyde. Groups 1 to 4 represent the negative control group, the extract control group, the ulcer control group, and the reference control group, respectively. The experimental groups are presented as groups 4–8. All values are expressed as mean  ±  standard error mean. Mean difference is significant at the *P* < 0.05 level (one-way between groups ANOVA with post hoc analysis). *Significant when compared with the group 3. ^#^Significant when compared with the group 4.
