# Corrigendum to “Acute Toxicity and Gastroprotective Role of* M. pruriens* in Ethanol-Induced Gastric Mucosal Injuries in Rats”

**DOI:** 10.1155/2018/1509057

**Published:** 2018-11-01

**Authors:** Shahram Golbabapour, Maryam Hajrezaie, Pouya Hassandarvish, Nazia Abdul Majid, A. Hamid A. Hadi, Noraziah Nordin, Mahmood A. Abdulla

**Affiliations:** ^1^Department of Biomedical Science, Faculty of Medicine, University of Malaya, 50603 Kuala Lumpur, Malaysia; ^2^Institute of Biological Sciences, Faculty of Science, University of Malaya, 50603 Kuala Lumpur, Malaysia; ^3^Department of Chemistry, Faculty of Science, University of Malaya, 50603 Kuala Lumpur, Malaysia; ^4^Department of Pharmacy, Faculty of Medicine, University of Malaya, 50603 Kuala Lumpur, Malaysia

The article titled “Acute Toxicity and Gastroprotective Role of* M. pruriens* in Ethanol-Induced Gastric Mucosal Injuries in Rats” [1] was found to contain figures from an earlier article by the same authors, Iza Farhana Ismail et al. [2], where Figure 3(b) is the same as Figure 2(a) in Iza Farhana Ismail et al. [2] and Figure 3(g) is the same as Figure 2(e) in Iza Farhana Ismail et al. [2], when rotated and flipped.

An institutional investigation by the University of Malaya found there was no system to index and file data and images to avoid mislabeling and mishandling, which led to errors and duplication of research data. The authors did not thoroughly check the manuscript before submission.

The authors said that the problems probably occurred during image labelling/embedding. Apart from mistakes in embedding some of the images, the rotation of the images was applied to consistently frame all of the images in the article (974185, horizontal orientation and 404012, vertical orientation).

Experimentally, the two articles had the same negative control group; therefore, 404012 Figure 2(a) and 974185 Figure 3(b) should have been corrected to 404012 Figure 2(a) and 974185 Figure 3(a), respectively, as they are both the negative control group, and 974185 Figure 3(a) should have been labelled 974185 Figure 3(b), showing the extract control group.

The editorial board recommended issuing a corrigendum.

## References

[1] Golbabapour S., Hajrezaie M., Hassandarvish P. (2013). Acute toxicity and gastroprotective role of *M. pruriens* in ethanol-induced gastric mucosal injuries in rats. *BioMed Research International*.

[2] Ismail I. F., Golbabapour S., Hassandarvish P. (2012). Gastroprotective activity of *Polygonum chinense* aqueous leaf extract on ethanol-induced hemorrhagic mucosal lesions in rats. *Evidence-Based Complementary and Alternative Medicine*.

