# Supplementary material for: Acute Toxicity and Gastroprotective Role of M. pruriens in Ethanol-Induced Gastric Mucosal Injuries in Rats
Source: Biomed Res Int. 2013 May 28;2013:974185. doi: 10.1155/2013/974185 (PMC3678452; doi:10.1155/2013/974185)
Supplement: Supplementary file 1 — suppl M. pruriens extract (<5 g/kg) in the acute toxicity test, on Sprague Dawley rats (6-8 weeks old, male and female), did not show any sign of toxicity in histological sections (H&E staining) of liver and kidney and in serum biochemical parameters. [file 974185.f1.docx]

# Figure S1: Histological sections of liver and kidney in the acute toxicity test (H&E staining 20×). Administration of *M. pruriens* extract does not show any abnormality in the histological sections of the liver (first row) and kidney (second row) in the acute toxicity test. Section from rats treated with CMC (A and D). Sections from rats treated with 2 g/kg (B and E) and 5 g/kg (C and F) *M. pruriens*.

**Figure S1**

C

A

B


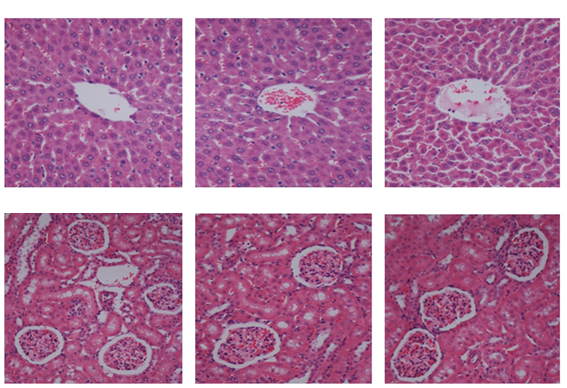


F

G

D
