# Supplementary material for: Acute Toxicity and Gastroprotective Role of M. pruriens in Ethanol-Induced Gastric Mucosal Injuries in Rats
Source: Biomed Res Int. 2013 May 28;2013:974185. doi: 10.1155/2013/974185 (PMC3678452; doi:10.1155/2013/974185)
Supplement: Supplementary file 2 [file 974185.f2.docx]

# Table S1. Acute toxicity test.

A. Effect of *M. pruriens* extract on the renal function tests of male rat in the acute toxicity study.

| Dose | Sodium  (mM/L) | Potassium  (mM/L) | Chloride  (mM/L) | CO_2_  (mM/L) | Anion gap  (mM/L) | Urea  (mM/L) | Creatinine  (µM/L) |
| --- | --- | --- | --- | --- | --- | --- | --- |
| Vehicle  (CMC) | 142.05 ± 0.58 | 4.89 ± 0.07 | 104.01 ± 0.87 | 24.85 ± 0.55 | 18.11 ± 0.58 | 5.50 ± 0.38 | 34.88 ± 2.38 |
| *M. pruriens*  (2 g/kg) | 142.03 ± 1.54 | 5.00 ± 0.06 | 106.13 ± 1.03 | 22.60 ± 1.19 | 18.17 ± 0.61 | 6.20 ± 0.50 | 33.67 ± 2.65 |
| *M. pruriens*  (5 g/kg) | 142.50 ± 0.86 | 4.95 ± 0.05 | 105.17 ± 0.58 | 23.96 ± 1.05 | 18.52 ± 0.45 | 5.63 ± 0.494 | 345.13 ± 4.90 |

Values expressed as the mean ± S.E.M. There were no significant differences between groups. The mean difference is significant at the *p*<0.05 level.

B. Effect of *M. pruriens* extract on the renal function tests of female rat in the acute toxicity study.

| Dose | Sodium  (mM/L) | Potassium  (mM/L) | Chloride  (mM/L) | CO_2_  (mM/L) | Anion gap  (mM/L) | Urea  (mM/L) | Creatinine  (µM/L) |
| --- | --- | --- | --- | --- | --- | --- | --- |
| Vehicle  (CMC) | 141.53 ± 1.410 | 4.66 ± 0.12 | 105.38 ± 0.67 | 23.38 ± 0.47 | 18.01 ± 0.36 | 7.95 ± 0.35 | 41.02 ± 2.75 |
| *M. pruriens*  (2 g/kg) | 141.00 ± 0.68 | 4.75 ± 0.16 | 105.53 ± 0.68 | 22.95 ± 0.45 | 17.13 ± 0.49 | 7.98 ± 0.24 | 42.00 ± 4.76 |
| *M. pruriens*  (5 g/kg) | 142.17 ± 1.47 | 4.54 ± 0.08 | 106.00 ± 0.63 | 21.98 ± 0.78 | 17.17 ± 0.49 | 8.31 ± 0.69 | 41.35 ± 2.15 |

Values expressed as the mean ± S.E.M. There were no significant differences between groups. The mean difference is significant at the *p*<0.05 level.

C. Effect of *M. pruriens* extract on the Liver function tests of male rat in the acute toxicity study.

| Dose | Total protein (g/L) | Albumin (g/L) | Globulin (g/L) | TB (µM/L) | CB  (µM/L) | AP  (IU/L) | ALT  (IU/L) | AST  (IU/L) | GGT  (IU/L) |
| --- | --- | --- | --- | --- | --- | --- | --- | --- | --- |
| Vehicle  (CMC) | 61.13  ± 1.08 | 9.16  ± 0.53 | 51.39  ± 1.33 | 2.13  ± 0.17 | 1.00  ± 0.00 | 153.07  ± 15.01 | 50.81  ± 1.71 | 173.53  ± 7.17 | 3.17  ± 0.16 |
| *M. pruriens*  (2 g/kg) | 58.97  ± 0.48 | 8.67  ± 0.33 | 49.73  ± 0.69 | 2.11  ± 0.15 | 1.00  ± 0.00 | 156.33  ± 15.24 | 48.02  ± 0.88 | 178.18  ± 5.84 | 3.67  ± 0.42 |
| *M. pruriens*  (5 g/kg) | 60.04  ± 1.03 | 9.70  ± 0.47 | 50.08  ± 1.05 | 2.08  ± 0.10 | 1.00  ± 0.00 | 155.17  ± 9.09 | 46.52  ± 1.76 | 175.87  ± 8.55 | 3.17  ± 0.17 |

Values expressed as the mean ± S.E.M. There were no significant differences between groups (Significant value at *p* < 0.05).

D. Effect of *M. pruriens* extract on the liver function tests of female rat in the acute toxicity study.

| Dose | Total protein (g/L) | Albumin (g/L) | Globulin (g/L) | TB (µM/L) | CB  (µM/L) | AP  (IU/L) | ALT  (IU/L) | AST  (IU/L) | GGT  (IU/L) |
| --- | --- | --- | --- | --- | --- | --- | --- | --- | --- |
| Vehicle  (CMC) | 64.38  ± 1.22 | 11.27  ± 0.17 | 53.37  ± 1.24 | 2.00  ± 0.00 | 1.00  ± 0.00 | 108.81  ± 6.33 | 43.05  ± 1.96 | 171.39  ± 6.86 | 3.77  ± 0.33 |
| *M. pruriens*  (2 g/kg) | 63.13  ± 1.11 | 11.00  ± 0.55 | 52.38  ± 1.29 | 2.00  ± 0.006 | 1.00  ± 0.00 | 101.33  ± 4.98 | 40.87  ± 2.73 | 172.27  ± 8.65 | 3.52  ± 0.51 |
| *M. pruriens*  (5 g/kg) | 65.01  ± 0.78 | 11.50  ± 0.45 | 53.08  ± 0.77 | 2.00  ± 0.00 | 1.00  ± 0.00 | 100.86  ± 5.36 | 44.15  ± 1.89 | 174.81  ± 5.81 | 3.00  ± 0.00 |

TB: Total bilirubin; CB: Conjugated bilirubin; AP: Alkaline phosphatase; ALT: Alanine aminotransferase; AST: Aspartate aminotransferase; GGT: γ-Glutamyltransferase. Values expressed as the mean ± S.E.M. There were no significant differences between groups (Significant value at *p* < 0.05).
